# Application of CAR-T cell therapy in orthopedics: From bone malignancies to non-malignant musculoskeletal disorders

**DOI:** 10.1016/j.jot.2026.101235

**Published:** 2026-10-10

**Authors:** Yimei Que, Xienilai Xie, Wei Xie, Yunzhou Zuo, Wang Chen, Zedong Xu, Changtian Gong, Xugui Li

**Affiliations:** a The Affiliated Hospital of Wuhan Sports University, Wuhan, China; b Hubei University of Chinese Medicine, Wuhan, China; c XiangNan University, Chenzhou City, Hunan Province, China; d Department of Orthopedics, Renmin Hospital of Wuhan University, Wuhan, 430060, China; e Center of Regenerative Medicine, Renmin Hospital of Wuhan University, Wuhan, 430060, China

**Keywords:** Autoimmune rheumatic diseases, Bone malignancies, Bone metastases, CAR T-cell therapy, Osteoarthritis, Tumour microenvironment

## Abstract

Chimeric antigen receptor T-cell (CAR-T) therapy has transformed hematologic oncology, but its application in orthopaedic diseases remains at an early stage. In bone malignancies, including osteosarcoma, chondrosarcoma, Ewing sarcoma, and bone metastases, antigen heterogeneity, limited trafficking and persistence, and the immunosuppressive skeletal microenvironment restrict efficacy. CAR-engineered approaches are also emerging in non-malignant disorders: CD19-directed CAR-T therapy has shown early clinical activity in refractory rheumatoid arthritis and other autoimmune rheumatic diseases, whereas senolytic CAR-T strategies for osteoarthritis remain preclinical. This review summarizes current evidence across malignant and non-malignant orthopaedic conditions, with emphasis on target selection, safety, and translational feasibility. Key barriers include antigen escape, impaired infiltration, limited persistence, local immunosuppression, manufacturing complexity, and on-target/off-tumour toxicity. Emerging strategies include multi-target receptors, chemokine receptor engineering, locoregional delivery, safety-control systems, and allogeneic or point-of-care manufacturing. Current evidence supports CAR-T and related CAR-engineered cells as potential complementary approaches for selected refractory orthopaedic conditions, although further translation requires stronger clinical evidence and improved biological and manufacturing strategies.

*The Translational Potential of this Article*: This review identifies disease-specific targets, barriers, and engineering priorities relevant to translating CAR-engineered cell therapies into orthopaedic practice. These considerations may inform preclinical validation and early-phase trials in refractory bone malignancies and selected non-malignant musculoskeletal disorders.

## Graphical abstract

**Figure:**
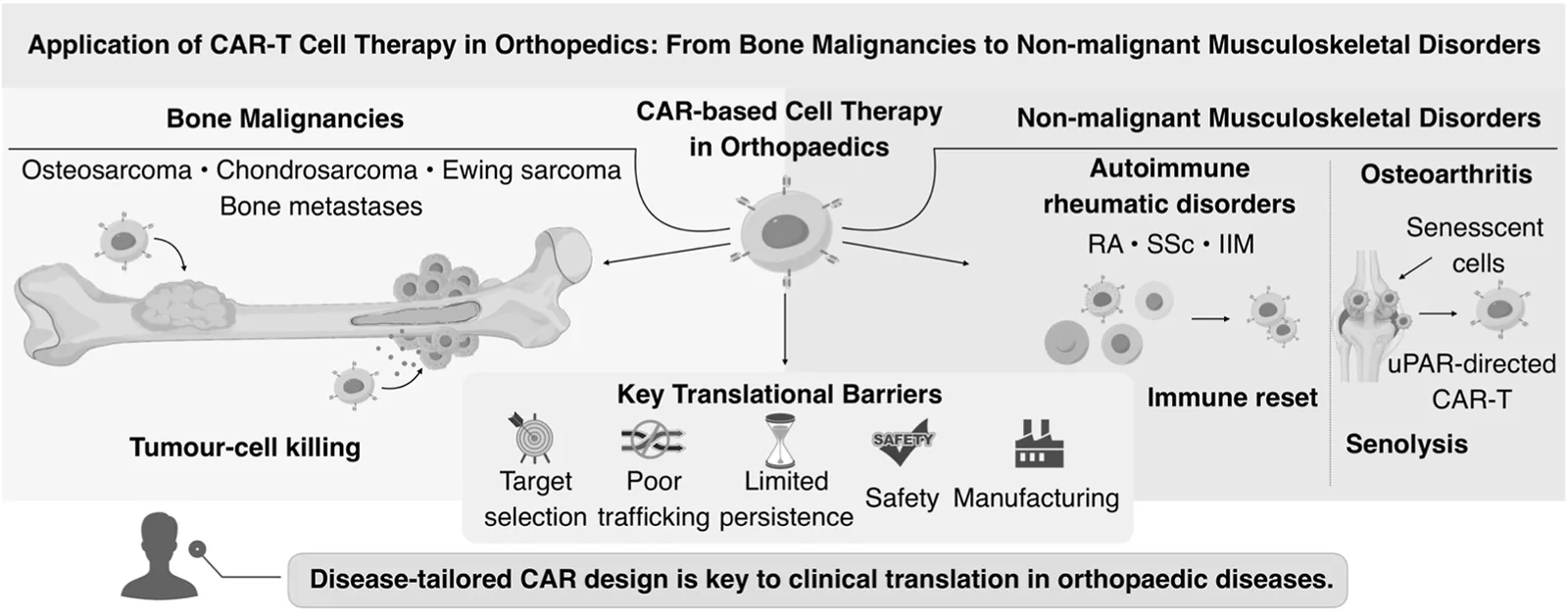

## 1 Introduction

Orthopedic diseases represent an urgent frontier for therapeutic innovation. Primary malignant bone tumors such as osteosarcoma, chondrosarcoma, and Ewing sarcoma, along with bone-dominant metastases from breast, prostate, lung, and renal cancers, remain associated with dismal outcomes, high recurrence rates, and debilitating skeletal complications [[1], [2], [3]]. Despite advances in surgery, radiotherapy, and multi-agent chemotherapy, the five-year survival rate for patients with metastatic osteosarcoma remains below 30% [4], and those with bone metastases from common carcinomas rarely survive beyond two years [3]. Likewise, immune-mediated skeletal disorders such as rheumatoid arthritis impose an enormous global burden and remain inadequately controlled by current disease-modifying agents [5]. Together, these challenges underscore a pressing need for more precise, durable, and mechanistically innovative therapeutic approaches.

Chimeric antigen receptor T-cell (CAR-T) therapy represents a paradigm shift in adoptive cellular immunotherapy [[6], [7], [8], [9]]. By genetically engineering patient-derived T cells to express synthetic receptors that directly recognize tumor-associated antigens, CAR-T cells bypass the need for antigen processing and presentation [7,9], enabling potent and specific cytotoxicity against malignant cells [7,8]. Incorporation of costimulatory domains such as CD28 or 4-1BB further enhances T-cell activation, persistence, and memory formation [10,11], allowing for long-term immune surveillance [[10], [11], [12]]. These features distinguish CAR-T therapy from conventional treatments and likely contribute to its success in hematologic malignancies, although their relevance in solid or skeletal diseases remains uncertain [7,12].

The transformative success of CAR-T therapy in hematologic malignancies demonstrates its potential [9]. CD19-directed CAR-T cells achieve complete remission rates of 70-90% in pediatric and adult B-cell acute lymphoblastic leukemia [13,14], while BCMA-targeted CAR-T cells induce objective response rates exceeding 70% in relapsed or refractory multiple myeloma [15,16]. Importantly, some patients maintain durable remissions lasting years [13,16,17] - outcomes rarely achieved with chemotherapy or hematopoietic stem-cell transplantation [12]. These milestones have firmly established CAR-T therapy as a clinical reality and catalyzed global efforts to extend its use to solid tumors [9,12]. Beyond tumour control, recent clinical data suggest that CAR-T therapy in multiple myeloma may also have secondary skeletal benefits, with a reduction in estimated fracture risk observed after treatment [18]. Although multiple myeloma is not a primary orthopaedic malignancy, this finding illustrates how effective control of marrow-based disease may translate into clinically relevant improvements in skeletal outcomes.

Importantly, the skeletal system is not immunologically inert [19,20]. Bone and joint homeostasis are governed by dynamic crosstalk between immune and osteogenic cells [19,20], and immune dysregulation underlies both tumor progression within the bone microenvironment [21,22] and the chronic inflammation driving degenerative and autoimmune diseases [23,24]. This immunological sensitivity suggests that immune-based interventions - long proven transformative in oncology [9,12] - may hold comparable promise in orthopedic disease. CAR-T cells, with their capacity for precise antigen-specific targeting, adaptability to heterogeneous disease landscapes, and potential for sustained immune surveillance, are uniquely poised to address these unmet clinical needs [25,26]. Beyond their proven cytotoxicity in hematologic malignancies, CAR-T cells could be harnessed to eliminate pathogenic stromal or senescent cell populations [27,28], remodel immunosuppressive niches [25,26], and restore immune equilibrium within skeletal tissues [19,28].

Nevertheless, translating the success of CAR-T therapy from hematologic malignancies to orthopedic indications remains a formidable challenge. In bone malignancies, profound antigenic heterogeneity, dense extracellular matrix and stromal architecture can hinder T-cell trafficking [29], while immunosuppressive microenvironments accelerate T-cell dysfunction [30]. Non-malignant musculoskeletal disorders present different challenges related to target selectivity, treatment durability, and acceptable safety thresholds. Overcoming these barriers will require strategic innovation in antigen selection, cell-engineering design, delivery strategies, and combinatorial approaches tailored to the unique biological context of skeletal tissues.

This review aims to critically evaluate the progress of CAR-T therapy in orthopedics, encompassing both malignant and non-malignant orthopedic disorders, which demand distinct CAR-T design philosophies. We summarize advances in antigen discovery, preclinical validation, and early-phase clinical translation, while dissecting the biological and translational barriers that currently limit efficacy and safety. Finally, we highlight emerging strategies-from dual-target CAR designs and chemokine receptor engineering to local delivery and immune-microenvironment modulation-and offer perspectives on how CAR-T therapy could evolve into a transformative therapeutic modality for orthopedic medicine. The conceptual landscape of CAR-T applications across malignant and non-malignant orthopedic diseases is summarized in Fig. 1. Key clinical and preclinical studies of CAR-engineered cell therapies across malignant and non-malignant musculoskeletal disorders are summarized in Table 1.

**Fig. 1:**
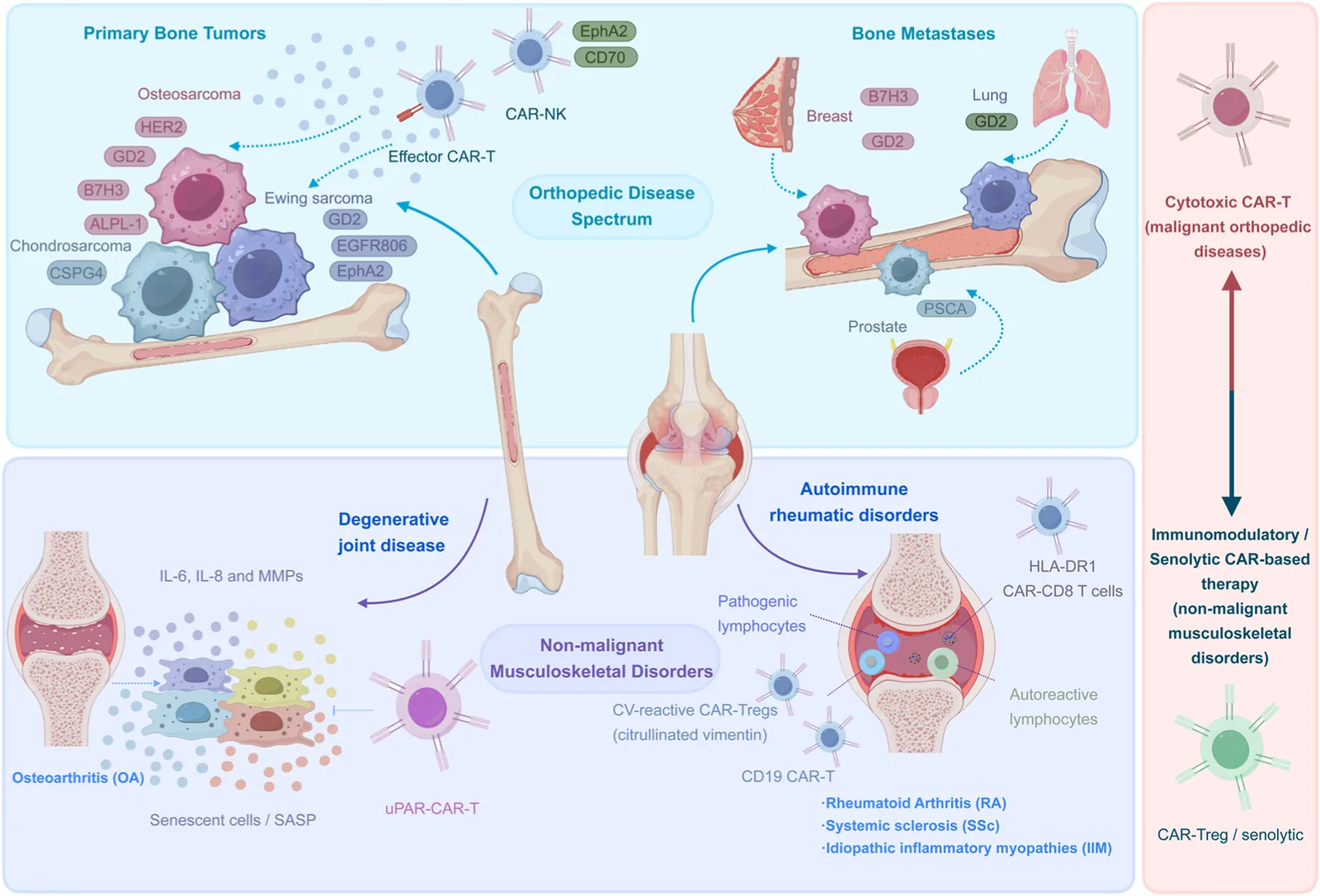
**Landscape of CAR-engineered cell therapies in orthopedic diseases.** Cytotoxic CAR-engineered cells target tumour-associated antigens in primary bone malignancies and bone metastases, whereas immune-regulatory approaches are being explored in autoimmune rheumatic disorders, including rheumatoid arthritis, systemic sclerosis, and idiopathic inflammatory myopathies. In osteoarthritis, CAR-based therapy follows a distinct senolytic rationale by targeting pathological senescent-cell populations. Emerging CAR-NK and CAR-M platforms provide complementary approaches to conventional CAR-T therapy.

**Table 1:** Clinical and preclinical landscape of CAR-engineered cell therapies in orthopaedic and musculoskeletal diseases.

Disease/indication	Target/strategy	Development stage	Key study/model	Key efficacy signal	Key safety signal	Key bottleneck	Reference(s)
Osteosarcoma/sarcoma	HER2 CAR-T	Phase I (NCT00902044)	Ahmed et al., 2015; Hegde et al., 2024, HER2-CAR-T clinical studies in advanced sarcoma	Stable disease in 4/19 in the earlier study; subsequent lymphodepletion-based Phase I testing confirmed feasibility with modest clinical activity	No dose-limiting toxicity; no treatment-related deaths reported	Limited expansion/persistence and trafficking in bone lesions	J Clin Oncol 2015; DOI:10.1200/JCO.2014.58.0225 Hegde et al., 2024 https://doi.org/10.1038/s43018-024-00749-6
Osteosarcoma/sarcoma	GD2 CAR-T	Phase I (NCT02107963)	Kaczanowska et al., GD2-CAR-T clinical study in solid tumors including osteosarcoma	Stable disease in 83% at day 28; all patients ultimately progressed; ∼30% maintained disease stability beyond 2 months	Low-grade CRS predominates; neurotoxicity uncommon (trial-reported)	Transient persistence; immunosuppressive myeloid/TME; antigen heterogeneity	Cancer Cell 2024; https://doi.org/10.1016/j.ccell.2023.11.011
Osteosarcoma	B7-H3 (CD276) CAR-T	Preclinical (orthotopic, spontaneous metastasis model)	Talbot et al., orthotopic OS model with spontaneous metastases	Dose-dependent control of primary tumor and metastases in vivo	Preclinical tolerability; off-tumor toxicity not prominent in mice	Clinical validation; trafficking and persistence in human bone lesions	Front Immunol 2021; https://doi.org/10.3389/fimmu.2021.691741
Osteosarcoma	CD166 (ALCAM) CAR-T	Preclinical (xenografts)	Wang et al., anti-CD166/4-1BB CAR-T in OS models	Tumor growth inhibition/regression in xenograft models; in vitro cytotoxicity vs OS lines	Potential on-target/off-tumor concern (CD166 expressed on normal tissues)	Target expression in healthy tissues limits therapeutic window	J Exp Clin Cancer Res 2019; https://doi.org/10.1186/s13046-019-1147-6
Osteosarcoma	ALPL-1 CAR-T (osteosarcoma-enriched isoform)	Preclinical (orthotopic primary & metastatic models)	Mensali et al., ALPL-1 as CAR target in OS	Robust cytotoxicity in vitro and antitumour activity in orthotopic OS models	No unexpected toxicity against HSCs/healthy tissues reported (preclinical)	Patient-to-patient expression variability; translational validation of isoform specificity	Nat Commun 2023; https://doi.org/10.1038/s41467-023-39097-x
Osteosarcoma (lung metastasis)	IL-11Rα CAR-T (SB transposon)	Preclinical	Huang et al., IL-11Rα-CAR T cells in OS lung metastasis models	Regression of established OS lung metastases in vivo	Preclinical study; clinical safety unknown	On-target/off-tumor risk and trafficking to metastatic niches	Cancer Res 2012; DOI:10.1158/0008-5472.CAN-11-2778
Osteosarcoma	NKG2D-ligand CAR-T	Preclinical	Fernández et al., 2017 and Hidalgo et al., 2026, NKG2D-CAR-T in osteosarcoma models	Antitumour activity in vitro and in mouse models, but more recent data showed limited in vivo tumour control despite preserved activity under hypoxia.	Broad ligand biology may increase off-tumor activation risk	Additional microenvironmental constraints beyond hypoxia; ligand heterogeneity	Clin Cancer Res 2017; DOI:10.1158/1078-0432.CCR-17-0075 Cancer immunology, immunotherapy. 2026 https://doi.org/10.1007/s00262-026-04319-w.
Osteosarcoma	OSM ligand CAR-T targeting OSMR/LIFR	Preclinical	Feinberg et al.; patient-derived and metastatic OS models	Antitumour activity across OSMR- or LIFR-expressing models	Preclinical only; clinical safety unknown	Receptor-expression heterogeneity; clinical validation	BMC medicine, 2026. https://doi.org/10.1186/s12916-026-04729-8.
Osteosarcoma	FOLR1 CAR-T	Preclinical (including PDX)	Choe et al., FOLR1 CAR-T cells in osteosarcoma cell-line and PDX models	Strong antitumour activity in osteosarcoma models, including PDX	Preclinical only; clinical safety not established	Patient-to-patient variation in antigen expression; clinical safety validation	Cancer Res Commun. 2025. DOI:10.1158/2767-9764.CRC-25-0086
Osteosarcoma	LRRC15 CAR-T	Preclinical (PDX and immunocompetent models)	O'Reilly et al., LRRC15-CAR-T cells in osteosarcoma PDX and immunocompetent models	Antitumour activity in multiple osteosarcoma models, including PDX and immunocompetent models	Preclinical only; clinical safety and normal-tissue effects remain to be established	Translation of preclinical efficacy; validation of normal-tissue safety	Clin Cancer Res. 2026 DOI:10.1158/1078-0432.CCR-24-2335
Osteosarcoma/sarcoma	EphA2 CAR-NK	Preclinical	Lam et al., mRNA-engineered CAR-NK	Enhanced cytotoxicity and in vivo antitumour activity	Preclinical only	Persistence and repeated dosing/engineering requirements	Clinical and translational medicine, 2025 https://doi.org/10.1002/ctm2.70140.
Osteosarcoma	CD70 CAR-NK + IL-15/dnTGFβR	Preclinical	Robbins et al., non-viral TcBuster-engineered CD70-CAR NK cells in osteosarcoma models	Enhanced cytotoxicity, persistence, and tumour homing in osteosarcoma models	Preclinical only; clinical safety not established	Clinical validation; durability and resistance to tumour-mediated immunosuppression	Mol Ther Oncol 2025; https://doi.org/10.1016/j.omton.2025.201119
Ewing sarcoma/paediatric bone tumors	EphA2 CAR-T	Preclinical	Hsu et al., EphA2-CAR-T cells in paediatric bone tumour models	Preclinical susceptibility of EphA2-positive paediatric bone tumors to CAR-T targeting	Preclinical only; clinical safety not established	Clinical validation; antigen-expression heterogeneity and tumour trafficking	Cancer Gene Ther. 2021 https://doi.org/10.1038/s41417-020-00221-4
Chondrosarcoma	CSPG4 CAR-T	Preclinical (in vitro)	Nota et al., Front Oncol 2022: CSPG4 TMA (n = 76) and CSPG4-CAR-T in-vitro cytotoxicity in CS cell lines.	Specific killing and cytokine release against CS cells in vitro	CSPG4 expression on pericytes/normal tissues raises off-tumor concern	Limited in vivo validation; safety window needs definition	PMID:36110930 https://doi.org/10.3389/fonc.2022.939166
Metastatic prostate cancer with bone disease	PSCA CAR-T	Phase I (NCT03873805)	Dorff et al., PSCA-CAR T in mCRPC	No objective responses by RECIST; ∼50% disease control, with PSA decline and bone-scan improvement in selected patients	DLT: cystitis (likely on-target/off-tumor); max grade 2 CRS; no high-grade neurotoxicity	Balancing lymphodepletion-driven activity with off-tumor toxicity; limited persistence	Nat Med 2024; https://doi.org/10.1038/s41591-024-02979-8
Autoimmune arthritis (RA-like models)	HLA-DR1 CAR-CD8 T cells targeting antigen-specific CD4^+^ T cells	Preclinical (HLA-DR1 transgenic mice)	Whittington et al., DR1-CII CAR-CD8 T cells in autoimmune arthritis model	Reduced disease severity and decreased CII-specific CD4 T cells in vivo	HLA restriction and potential off-target immune perturbation	Personalization (HLA/autoantigen) and manufacturability	J Immunol 2022; https://doi.org/10.4049/jimmunol.2100643
Rheumatoid arthritis	CD19 CAR-T	Early clinical	Lidar et al.; polyrefractory RA	Substantial clinical improvement reported in refractory RA	Early clinical experience; small patient numbers	Limited cohort size; durability and generalisability remain uncertain	Annals of the rheumatic diseases, 2025. https://doi.org/10.1136/ard-2024-226437.
Systemic sclerosis/idiopathic inflammatory myopathies	CD19 CAR-T	Early clinical/case series	Müller et al., CD19 CAR-T in severe autoimmune disease (IIM and SSc)	All 3 IIM patients achieved ACR–EULAR major clinical response; all 4 SSc patients showed reduced EUSTAR activity, with withdrawal of immunosuppression	Manageable in reported cohort	Small, highly selected cohorts; long-term durability	The New England journal of medicine, 2024. https://doi.org/10.1056/NEJMoa2308917.
Severe myositis/diffuse systemic sclerosis	Allogeneic CD19 CAR-T	Early clinical	Wang et al., allogeneic CD19 CAR-T therapy in severe myositis and diffuse systemic sclerosis	Rapid B-cell depletion with clinical responses and deep remission reported in the small cohort	No severe treatment-related toxicity reported in the small cohort	Very small cohort; allogeneic persistence, rejection and broader clinical validation	Cell. 2024; https://doi.org/10.1016/j.cell.2024.06.027
Osteoarthritis (senolytic rationale)	uPAR-directed senolytic CAR-T	Preclinical (senescence models; not OA-specific)	Amor et al., uPAR-CAR T as cellular senolytic	Clearance of uPAR + senescent cells and improvement of senescence-associated pathology	Preclinical; translation to chronic diseases requires safety validation	OA-specific target validation; preservation of beneficial transient senescent cells; local delivery and safety	Nature 2020; https://doi.org/10.1038/s41586-020-2403-9

Abbreviations: ALCAM, activated leukocyte cell adhesion molecule; CAR-NK, chimeric antigen receptor natural killer cell; CAR-T, chimeric antigen receptor T cell; CII, type II collagen; CRS, cytokine release syndrome; CS, chondrosarcoma; CTC, circulating tumour cell; DCR, disease control rate; DL, dose level; DLT, dose-limiting toxicity; dnTGFβR, dominant-negative transforming growth factor-β receptor; EphA2, ephrin type-A receptor 2; FOLR1, folate receptor alpha; HLA, human leukocyte antigen; HSC, hematopoietic stem cell; IIM, idiopathic inflammatory myopathies; LD, lymphodepletion; LIFR, leukemia inhibitory factor receptor; LRRC15, leucine-rich repeat-containing protein 15; mCRPC, metastatic castration-resistant prostate cancer; NTX, neurotoxicity; OA, osteoarthritis; ORR, objective response rate; OS, overall survival; OS (disease), osteosarcoma; OSMR, oncostatin M receptor; PDX, patient-derived xenograft; PFS, progression-free survival; PSCA, prostate stem cell antigen; RA, rheumatoid arthritis; SB, Sleeping Beauty; SSc, systemic sclerosis; TMA, tissue microarray; TME, tumour microenvironment; uPAR, urokinase-type plasminogen activator receptor.

## 2 CAR-T therapy in bone tumors

### 2.1 Osteosarcoma (OS)

Osteosarcoma (OS) remains the most extensively studied orthopedic malignancy for CAR-T therapy, with several antigens explored in both preclinical and clinical contexts. Among these, HER2 and GD2 have progressed to clinical evaluation, providing critical insights into the feasibility of CAR-T therapy for OS. In a Phase I/II study of HER2-directed CAR-T cells (NCT00902044), 19 patients with sarcomas, including 16 with OS, were treated; four achieved stable disease lasting from 12 weeks to 14 months, with a median overall survival of 10.3 months [31]. More recently, a Phase I trial demonstrated that autologous HER2-CAR-T cells, administered after lymphodepletion, were safe and associated with disease stabilization in selected advanced sarcoma patients, although efficacy remained modest [32]. These findings highlight the therapeutic potential of HER2 targeting, consistent with HER2's established oncogenic role [33] and its prognostic significance in OS [34], yet clinical activity of HER2-directed antibody-drug conjugates (e.g., trastuzumab deruxtecan) has so far been limited in recurrent osteosarcoma [35]. Importantly, safety remains a concern, as exemplified by a prior case report of fatal toxicity with ERBB2-directed CAR-T therapy [36], underscoring the need for careful dosing and target validation.

GD2-CAR-T therapy, by contrast, has shown higher initial rates of disease control but limited long-term benefit. In a Phase I trial (NCT02107963) involving 15 patients (12 OS), 83% achieved stable disease at day 28; however, all eventually progressed, and only 30% maintained disease stability beyond two months [37]. While HER2 CAR-T has occasionally induced more durable stabilization, GD2 CAR-T is associated with lower toxicity, generally limited to grade 1 CRS, and no significant neurotoxicity, making it more tolerable in clinical practice [37]. Recent work has further identified immune determinants of CAR-T expansion in GD2-treated patients, linking treatment outcome to host immune fitness [37]. Additionally, bispecific antibody-armed T cells targeting GD2 and CD3 are under investigation for OS, demonstrating synergistic cytotoxicity in refractory disease [38]. Notably, the comparison between HER2 and GD2 targeting highlights a persistent trade-off between efficacy and safety, which remains unresolved in current clinical development: These non-comparative early studies suggest that HER2-directed therapy may occasionally achieve longer disease stabilization but carries target-related safety concerns, whereas GD2-CAR-T has shown a favourable acute safety profile but limited durability.

Beyond these clinically tested antigens, novel targets are expanding the therapeutic repertoire. NKG2D ligand-targeting CAR-T cells have demonstrated antitumour activity in preclinical sarcoma models, providing a heterogeneity-tolerant targeting strategy [39]. In contrast, a recent NKG2D-CAR-T study showed strong in vitro cytotoxicity but limited tumour control in vivo; hypoxia alone did not explain this discrepancy, indicating that additional microenvironmental factors may restrict CAR-T activity [40]. B7-H3 has emerged as a highly expressed pan-sarcoma antigen, and CAR-T cells directed against it induced regression of established xenografts in preclinical models [41,42], including orthotopic osteosarcoma models [43]. ALPL-1, recently validated as an OS-associated antigen, showed selective cytotoxicity against metastatic OS, particularly lung lesions, with minimal off-tumor toxicity [44]. Similarly, ALCAM (CD166)-CAR-T cells were capable of killing OS cell lines in vitro and inhibiting tumor growth in vivo, with efficacy correlating to antigen density [45]. Other promising candidates include FAP [46] and CSPG4 [47], both implicated in tumor progression and metastatic behavior. Notably, CSPG4 has also been suggested as a combinatorial target for OS, though its expression in other mesenchymal tissues requires further evaluation. More recent studies have expanded the range of candidate targets in osteosarcoma. An OSM ligand-based CAR capable of recognising OSMR- or LIFR-expressing tumour cells showed activity in patient-derived and metastatic models, providing a potential strategy to broaden antigen coverage [48]. FOLR1-and LRRC15-directed CAR-T cells also showed encouraging activity in osteosarcoma models, including patient-derived xenografts and, for LRRC15, immunocompetent models [49,50]. These approaches remain preclinical and require clinical validation. CAR engineering has also been extended to NK cells in osteosarcoma. EphA2-CAR-NK cells showed enhanced cytotoxicity and in vivo antitumour activity in osteosarcoma models, while CD70-CAR-NK cells incorporating IL-15 and a dominant-negative TGF-β receptor showed improved cytotoxicity, persistence, and tumour homing [51,52].

In summary, OS represents both the most fertile ground and one of the greatest challenges for CAR-T translation in orthopedics. Clinical experience with HER2 and GD2 underscores the trade-offs between efficacy and safety, while preclinical studies of B7-H3, ALPL-1, and CSPG4 point to the promise of novel antigens with greater specificity. Future target selection should therefore consider more than antigen expression alone. Priority should be given to targets that combine sufficiently broad and dense tumour expression with limited expression in essential normal tissues, low intra- and inter-tumoural heterogeneity, and biological relevance to tumour growth or metastasis. The maturity of supporting evidence should also be considered, because a biologically attractive target may still be far from clinical translation if safety and in vivo efficacy have not been adequately established. Based on these considerations, HER2 and GD2 currently have the advantage of clinical experience, whereas B7-H3, ALPL-1, and CSPG4 remain promising but require further clinical validation and safety assessment. A comparative summary of these targets is provided in Supplementary Table S1. In addition, IL-11Rα-directed CAR-T cells have demonstrated regression of established osteosarcoma lung metastases in preclinical models, further supporting the feasibility of targeting metastatic niches in OS [53]. However, antigen heterogeneity and the profoundly immunosuppressive OS microenvironment continue to limit durable responses. Future strategies will require rational antigen prioritization, incorporation of combinatorial or logic-gated CAR designs [54], and approaches to enhance persistence and infiltration, including modulation of myeloid-derived suppressor cells [55]. Only through integration of these innovations can CAR-T therapy be advanced toward meaningful clinical benefit in OS.

### 2.2 Chondrosarcoma

Chondrosarcoma is largely refractory to systemic therapy, with surgery remaining the primary curative option. Conventional chemotherapy provides limited benefit in most subtypes [56,57]. In IDH1-mutant conventional chondrosarcoma, ivosidenib has shown durable disease stabilization with a median PFS of ∼5.6 months, with objective responses uncommon [58]. Anti-angiogenic therapy with pazopanib yielded a 16-week disease control rate of 43% with rare objective responses in a phase II study [59]. For anatomically challenging disease (notably skull-base chordoma/chondrosarcoma), carbon-ion radiotherapy achieves high long-term local control in meta-analyses, but does not address systemic progression [60]. Overall, outcomes for patients with advanced or metastatic disease remain poor, with median survival often below two years.

Against this backdrop, CSPG4 has emerged as the most promising CAR-T target. Immunohistochemical studies demonstrate frequent and strong CSPG4 expression in chondrosarcoma [61,62], with functional analyses showing its role in tumor migration and invasion [62]. Importantly, CSPG4 expression is limited in normal tissues, making it an attractive candidate for tumor-specific targeting [61]. Furthermore, evidence from other cancers highlights CSPG4 as a druggable antigen, with antibody-based approaches showing activity in triple-negative breast cancer [63] and high expression correlating with poor prognosis in breast tumors [64]. Preclinical work confirms that CSPG4-CAR-T cells can selectively lyse CSPG4-positive chondrosarcoma cells, offering proof-of-principle for this strategy [61].

However, translation remains premature: existing evidence is confined to in vitro and xenograft models, and possible low-level expression in perivascular or stromal compartments raises safety concerns. Compared with systemic options like ivosidenib or pazopanib, which provide modest but clinically validated benefits, CAR-T therapy for chondrosarcoma is still conceptual. Its advancement will require rigorous antigen safety profiling, strategies to overcome antigen heterogeneity and the immunosuppressive microenvironment, and early-phase trials to test whether CSPG4-CAR-T can outperform or complement existing treatments.

### 2.3 Ewing sarcoma

Ewing sarcoma (ES) is the second most common primary bone malignancy in children and adolescents [65]. Despite intensive multimodal therapy, outcomes remain unsatisfactory for advanced disease: contemporary series report 5-year survival <30% in metastatic ES and ∼10-15% after recurrence, highlighting the need for novel strategies [65,66]. Standard front-line chemotherapy (e.g., VDC/IE or related backbones) achieves 3-year event-free survival ≈70-75% in standard-risk cohorts, but these figures fall sharply in metastatic/relapsed settings [67].

CAR-T evidence in ES remains early and largely extrapolated from pan-sarcoma programs, with the most mature signal around GD2. In the Phase I study NCT02107963, GD2-CAR-T was feasible and safety-dominant; across enrolled sarcomas (including osteosarcoma and limited ES), day-28 disease control was common (stable disease dominated), but durability was poor with progression in subsequent weeks to months [37]. Correlative analyses linked expansion/clinical control to host immune fitness (e.g., CXCR3+ monocytes) rather than dose alone, underscoring microenvironmental and systemic determinants of response [37].

Beyond GD2, EGFR806 (a tumor-selective conformational epitope of EGFR) is under prospective evaluation in children/young adults with relapsed/refractory solid tumors including ES (NCT03618381; Phase I), with results pending [68,69]. NKG2D-ligand CARs showed broad in-vitro activity across ES family cell lines and antitumour effects in xenografts, offering a heterogeneity-tolerant approach [70]. EphA2, a driver of ES invasion/metastasis, has demonstrated preclinical susceptibility to CAR-T targeting and is being refined for pediatric bone tumors [71,72]. In parallel, IGF-1R-directed strategies—while biologically compelling in ES—have not produced consistent clinical benefit as single agents; combinations with mTOR inhibition appear more active than IGF-1R blockade alone, but remain investigational [[73], [74], [75], [76]].

Head-to-head with current ES care, CAR-T has not yet surpassed standard chemotherapy or salvage regimens on hard endpoints: there are no confirmed objective responses or durable remissions specific to ES in early CAR-T trials to date, in contrast to front-line VDC/IE outcomes in standard-risk disease [37,77]. Taken together, available data suggest that transient disease control without durability is the prevailing CAR-T pattern in ES, likely constrained by antigen heterogeneity, poor persistence, and an immunosuppressive microenvironment. Future development will require rational antigen selection (e.g., EGFR806/EphA2/NKG2D-L), microenvironment modulation, and combination strategies (e.g., with targeted/anti-angiogenic agents or myeloid reprogramming) to test whether CAR-T can match or exceed current systemic benchmarks in relapsed ES.

### 2.4 Bone metastases

Bone metastases are a common and devastating complication of advanced cancers, particularly prostate, breast, lung, and renal cell carcinomas [78]. They are associated with skeletal-related events (SREs)—including pathological fractures, spinal cord compression, and severe pain—that markedly impair quality of life and shorten survival [78]. Current treatments focus largely on palliation. Radiotherapy provides local control and symptom relief, while bone-modifying agents such as bisphosphonates or denosumab reduce the risk of SREs by ∼30-40% [[79], [80], [81]]. Radium-223 in metastatic castration-resistant prostate cancer (mCRPC) prolongs median overall survival to ∼14 months and delays skeletal events to ∼15 months [82]. Nevertheless, systemic disease control remains poor, with survival after bone-dominant metastasis often limited to 2-3 years in prostate cancer and <2 years in breast or lung cancers [83,84]. Notably, immunotherapy responses are site-dependent: meta-analyses in NSCLC show that patients with bone metastases derive significantly less benefit from checkpoint inhibitors compared to other metastatic sites [85], highlighting the bone microenvironment as a particularly resistant niche.

CAR-T therapy has recently been translated into bone metastatic disease, with PSCA (prostate stem cell antigen) being the most advanced target. PSCA is highly expressed in prostate cancer and enriched in osseous lesions [86,87]. In a first-in-human Phase I trial (NCT03873805), patients with heavily pretreated mCRPC, most with extensive bone metastases, received PSCA-CAR-T following lymphodepletion. Updated data reported at Nature Medicine in 2024 showed that although no objective responses by RECIST were achieved, ∼50% of patients attained disease control, with cases of prostate-specific antigen (PSA) decline and bone scan improvements [88]. Circulating tumor cell reductions were observed in ∼40% of evaluable patients [88], and toxicity was manageable, primarily low-grade CRS. Importantly, CAR-T cells trafficked to bone marrow and metastatic lesions, establishing proof-of-concept for CAR-T infiltration into skeletal disease [88].

Other targets are under active study. B7-H3-directed CAR-T has demonstrated potent activity in xenograft models of prostate and breast cancer bone metastases [42]. Other CAR targets may also have relevance to cancers that commonly involve bone. For example, GD2-directed CAR-T cells have shown preclinical activity in breast and lung cancer models, although their efficacy specifically against established bone metastases remains to be determined [89,90]. Beyond surface antigens, strategies exploiting NKG2D ligands or chemokine axes such as CXCR4/SDF-1 have been proposed to enhance T-cell homing and overcome bone marrow immune exclusion [91,92].

Compared with existing therapies, CAR-T remains in its infancy. Whereas bisphosphonates, denosumab, and radium-223 provide reproducible improvements in skeletal event rates and modest survival benefits [78,82], CAR-T so far has offered only stabilization without tumor regression. However, CAR-T's unique ability to traffic to marrow and dynamically adapt to antigen expression suggests it could eventually complement or surpass current strategies. The distinctive challenge in bone metastases lies not only in antigen heterogeneity but also in the specialized bone microenvironment, which is rich in stromal cells, ECM regulators such as SERPINH1 [93], and immunosuppressive signals (e.g., VEGF-mediated vascular dysfunction [94], NF-κB suppression [95], abnormal macrophage polarization [96]). These features may explain why immune checkpoint inhibitors underperform in bone lesions [97] and why CAR-T must be tailored with bone-specific engineering.

In summary, CAR-T therapy for bone metastases is at a proof-of-concept stage. PSCA-CAR-T has shown safety, trafficking, and signs of bioactivity in prostate cancer with bone lesions [88], but durable regressions are lacking. Future directions will require microenvironment-informed strategies—such as combining CAR-T with anti-VEGF or vascular normalization [98], CXCR4 engineering to enhance bone-marrow homing [91], or synergistic modalities like engineered macrophages [99]. Unlike osteosarcoma or chondrosarcoma, where target discovery dominates, the bottleneck in bone metastases may lie in remodeling the immunosuppressive skeletal niche, making it a distinct frontier for CAR-T innovation.

## 3 CAR-T therapy in non-malignant musculoskeletal disorders

### 3.1 Rheumatoid arthritis (RA)

Rheumatoid arthritis (RA) is a systemic autoimmune disease affecting roughly 1% of adults, with U.S. prevalence rising over 2005-2018 and clear disparities by sex, race/ethnicity, obesity, and socioeconomic status [5]. Conventional therapy with methotrexate (MTX), biologics (TNF/IL-6R/CTLA-4), and JAK inhibitors has transformed care, yet ∼30-40% of patients do not achieve durable remission and relapse on tapering remains common [100,101]. Response heterogeneity is substantial: pharmacogenetic studies implicate MTHFR, ABCB1, and SLCO1B1 variants in MTX efficacy and toxicity, supporting genotype-informed dosing in difficult-to-treat patients [102]. B-cell depletion is also imperfect; a single rituximab dose markedly reduces circulating B cells but fails to clear B cells in secondary lymphoid organs, altering phenotype rather than fully eliminating disease-relevant niches [103].

Against this backdrop, CAR-based strategies in RA aim to restore immune tolerance with greater precision than systemic immunosuppression. Proof-of-concept studies have developed citrullinated vimentin (CV)-reactive CAR-Tregs, demonstrating antigen-specific recognition/activation in RA synovial fluid and CV-expressing targets, while in vivo validation and broader HLA applicability remain key next steps [104,105]. In parallel, cytotoxic approaches provide a complementary path: a ‘universal’ anti-FITC CAR-T platform can be redirected by FITC-labelled citrullinated peptide epitopes to eliminate autoreactive B-cell subsets from patients with RA in vitro [106]. Feasibility and safety of Treg transfer in autoimmunity are supported by a first-in-human SLE case, lending translational confidence to a tolerance-reprogramming approach [107]. Complementary cytotoxic CAR-T concepts target pathogenic lymphocytes directly. Universal CAR-T cells selectively eliminate autoreactive B cells from RA patients in vitro, reducing autoantibody production [106], and HLA-DR1-engineered CD8^+^ CAR-T cells ablate antigen-specific CD4^+^ effectors and prevent arthritis development in murine models [108]. These mechanisms directly address limitations of anti-CD20 therapy in lymphoid tissues [103] and may synergize with biologics in induction or maintenance. Recent clinical reports of CD19-CAR-T therapy in refractory RA have shown substantial disease improvement, extending the evidence for CAR-T in RA beyond preclinical studies, although patient numbers remain small [109].

Enabling technologies can further tilt the risk-benefit balance. Immunomodulatory nanomedicines enhance synovial accumulation and retention, lower systemic exposure, and permit co-delivery of immune-tuning payloads, offering a route to combine cell therapy with localized, steroid-sparing control of joint inflammation [110,111]. Strategic priorities therefore include stabilizing the Treg phenotype under inflammatory stress [112,113], broadening beyond HLA-DR1 restrictions [105], and employing transient or switchable CARs and intra-articular delivery to balance persistence with safety [[114], [115], [116]]. CAR-M has also been proposed as an alternative approach for rheumatic diseases because macrophages are directly involved in synovial inflammation and tissue damage. However, unlike CAR-T approaches, its application in RA remains conceptual and preclinical, and disease-specific efficacy has yet to be demonstrated [117]. As prevalence rises and population heterogeneity widens [5], pharmacogenetic stratification [118], B-cell niche targeting [103,106,108], and nano-enabled modulation [110,111] together position RA as a lead indication for CAR-T-based tolerance engineering in autoimmune rheumatology.

### 3.2 Other autoimmune rheumatic disorders with musculoskeletal involvement

Beyond RA, clinical experience with CD19-directed CAR-T therapy has also emerged in systemic sclerosis (SSc) and idiopathic inflammatory myopathies (IIM), both of which can involve substantial musculoskeletal dysfunction. In a case series of 15 patients with severe autoimmune disease, all three patients with IIM achieved an ACR-EULAR major clinical response, while all four patients with SSc showed reduced EUSTAR disease activity after CD19 CAR-T treatment, with discontinuation of immunosuppressive therapy in all patients [119]. Subsequent studies in diffuse SSc have reported improvement in disease activity and fibrotic manifestations after CD19 CAR-T therapy [120]. Allogeneic CD19 CAR-T cells have also produced clinical responses in refractory immune-mediated necrotising myopathy and diffuse SSc, providing early evidence that the approach may extend beyond autologous products [121]. These findings broaden the potential application of CAR-T therapy in rheumatic disease, although current evidence is based on small, highly selected patient cohorts and longer follow-up is still needed.

### 3.3 Osteoarthritis (OA)

Beyond autoimmune joint disease, CAR-based approaches have also been explored in degenerative conditions such as OA, although the therapeutic rationale is different. Osteoarthritis (OA) is a multifactorial degenerative joint disorder rather than a primarily immune-mediated disease. Nevertheless, low-grade inflammation, synovial immune responses, and cellular senescence contribute to disease progression. Current treatments remain largely symptomatic, and effective disease-modifying therapies are limited [122,123].

Cellular senescence provides the main rationale for exploring CAR-T therapy in OA [124,125]. Senescent chondrocytes and synovial cells can accumulate in diseased joints and contribute to chronic inflammation through the senescence-associated secretory phenotype (SASP) [124,125]. In a murine proof-of-concept study, uPAR-directed CAR-T cells reduced senescent-cell burden and improved senescence-associated pathology, supporting the feasibility of using CAR-T cells as a senolytic strategy rather than as conventional immunotherapy [28,126].

A major challenge is selectivity. Senescent cells are not uniformly harmful, and transient senescence contributes to wound healing and tissue repair [127]. Future senolytic CAR-T strategies will therefore need to distinguish persistent, pathogenic senescent cells from temporary repair-associated populations, potentially through more selective antigen combinations and transient or locally delivered CAR designs. At present, uPAR-CAR-T should therefore be regarded as a proof-of-concept approach for OA rather than an established therapeutic strategy [28].

## 4 Challenges of CAR-T therapy in orthopedics

While CAR-T therapy has achieved remarkable progress in hematologic malignancies, its translation to malignant and non-malignant orthopedic disorders presents unique hurdles. One of the most immediate barriers is the cost and complexity of cell manufacturing. Unlike hematologic malignancies, where centralized production pipelines already exist, the relatively low incidence and heterogeneity of bone tumors such as osteosarcoma or Ewing sarcoma make individualized production disproportionately expensive [128]. In a conventional autologous model, each patient requires a separate cell collection, manufacturing run, quality-control process, and product release, making it difficult to spread fixed production costs across a small patient population. This is particularly relevant for heavily pretreated sarcoma patients, in whom T-cell collection and expansion may also be more challenging. Current estimates in hematology exceed USD 350,000-450,000 per infusion [129], and the absence of economies of scale may further limit the feasibility of this approach in rare orthopedic malignancies. Progress in allogeneic “off-the-shelf” CAR-T platforms may therefore be particularly relevant, as a single manufacturing batch could potentially be used for multiple patients and treatment could be initiated without waiting for individualized cell production [130]. However, this approach also introduces additional challenges, including host-versus-graft rejection, graft-versus-host disease, limited persistence, and the need for further genome engineering. Therefore, allogeneic products should not yet be considered a universal replacement for autologous CAR-T therapy in bone sarcoma. Point-of-care manufacturing provides a different possible solution by retaining an autologous product while shortening the production chain and reducing transport and cryopreservation requirements. For rare sarcomas, this model may be most practical in specialized centres where the same manufacturing platform can be shared across several cellular therapy programmes. Thus, allogeneic and point-of-care manufacturing should currently be viewed as complementary approaches rather than absolute requirements. Their relative value is likely to depend on the clinical setting: individualized autologous therapy may remain feasible for selected patients with aggressive, treatment-refractory sarcomas, whereas wider application of CAR-based therapies to common chronic disorders such as osteoarthritis or rheumatoid arthritis would require substantially lower manufacturing costs and greater production capacity.

While manufacturing cost and access remain major translational hurdles, bone-specific biological barriers further constrain CAR-T trafficking, function and persistence (Fig. 2). Antigen heterogeneity is another critical obstacle. Targets explored in orthopedics-including GD2, HER2, B7-H3, and CSPG4-exhibit variable expression not only across tumor types but even within individual lesions [42,61,131]. In osteosarcoma, heterogeneous HER2 and GD2 expression has been linked to immune escape and relapse after CAR-T infusion [32,132]. In chondrosarcoma, CSPG4 is highly expressed in some subtypes but absent in others [61], complicating patient selection. This heterogeneity is compounded by the bone microenvironment itself: stromal cells, osteoblasts, and extracellular matrix components can physically shield tumor cells and dynamically modulate antigen expression [133]. Dual-target CARs, bispecific constructs, and synthetic Notch (synNotch) circuits have emerged as promising strategies to overcome this complexity [54,134,135].

**Fig. 2:**
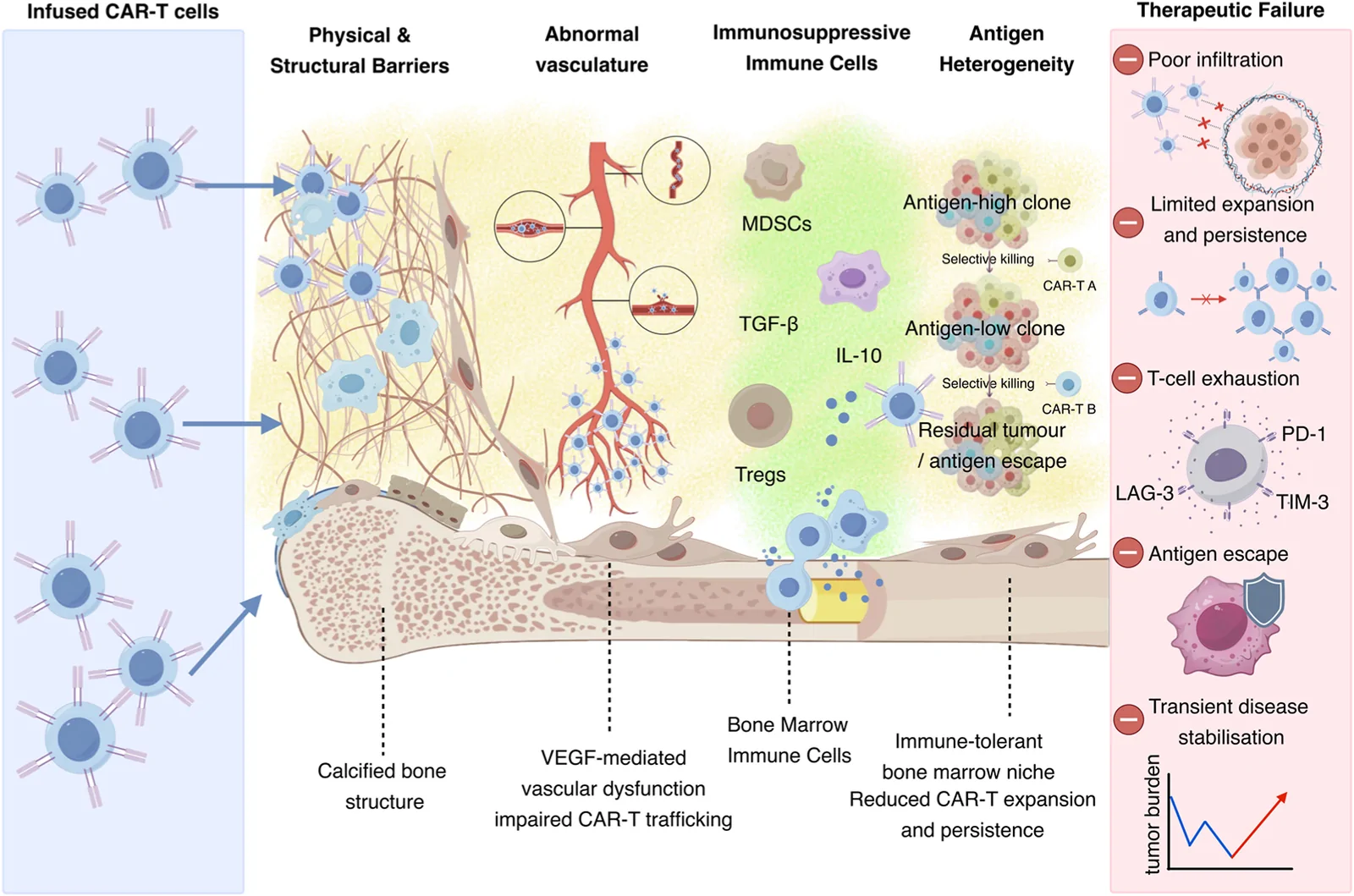
**Barriers to CAR-T cell activity in the bone tumor microenvironment.** Following infusion, CAR-T cells encounter multiple barriers within the bone microenvironment that limit their trafficking and function. Physical and structural constraints, including the dense extracellular matrix and calcified bone architecture, restrict infiltration into tumor sites. In parallel, abnormal and VEGF-driven vasculature impairs effective CAR-T cell trafficking within bone tissue. Within the bone marrow, an immunosuppressive milieu further constrains CAR-T cell activity. Myeloid-derived suppressor cells, regulatory T cells and inhibitory cytokines, including TGF-β and IL-10, reduce T-cell expansion and effector function. In addition, heterogeneity of tumor-associated antigen expression enables immune escape and contributes to incomplete tumor elimination. These combined factors lead to limited CAR-T cell expansion, functional exhaustion characterized by upregulation of inhibitory receptors such as PD-1, LAG-3 and TIM-3, and ultimately transient disease control.

The limited expansion and persistence of CAR-T cells in the context of orthopedic tumors also undermines efficacy. In sarcoma trials, CAR-T expansion has been modest and responses transient. Bone marrow and metastatic niches are particularly hostile environments, characterized by poor vascularization and abundant stromal barriers that restrict infiltration. Approaches such as CXCR4 or CCR2b chemokine receptor engineering to guide T cells into bone marrow and metastatic foci may prove especially relevant for bone-localized disease [136]. Recent work suggests that site-specific integration of CAR constructs into the PDCD1 locus can improve T-cell persistence and resistance to exhaustion [137], offering a potential solution to the problem of short-lived responses in bone lesions. The predominance of disease stabilization rather than objective tumour regression in early HER2-, GD2-, and PSCA-CAR-T trials suggests that current CAR-T cells can exert antitumour activity, but that this activity is often insufficient in depth or duration. This is unlikely to reflect CAR signalling strength alone. Heterogeneous or low antigen expression may leave part of the tumour unrecognised, while limited CAR-T expansion, poor trafficking, and suppression within the bone microenvironment further reduce effective tumour killing. Improving objective responses will therefore require better selection of patients with sufficient antigen expression, strategies to reduce antigen escape, and approaches that improve CAR-T access, persistence, and function within skeletal lesions.

Locoregional delivery may partly address the trafficking barrier by increasing CAR-T exposure at skeletal or joint sites while limiting systemic exposure. Intra-osseous or intra-articular administration may therefore be useful for anatomically accessible lesions [32,88,138]. However, local administration also has practical limitations. Injection into a lesion does not necessarily result in uniform CAR-T distribution, particularly in large or heterogeneous bone tumors containing necrotic areas, dense extracellular matrix, or poorly accessible compartments. Similarly, intra-articular delivery may not ensure even exposure across the entire synovium. Local delivery also does not by itself solve the problem of CAR-T persistence. Cells delivered directly into bone or joint tissue remain exposed to the same immunosuppressive signals that limit CAR-T function after systemic administration, and their local expansion and retention may therefore still be limited [138]. Repeated administration may partly compensate for short persistence, but this is more practical for accessible joints than for intra-osseous or intratumoral injection, which may require repeated image-guided procedures. In addition, local delivery is less suitable for patients with multiple bone lesions or extra-skeletal metastases, where systemic administration has the advantage of reaching several disease sites after a single infusion. Therefore, locoregional delivery is more likely to complement rather than replace systemic CAR-T administration.

The tumor microenvironment (TME) in bone lesions imposes additional challenges. Osteosarcoma and metastatic bone disease are enriched in TGF-β, VEGF, myeloid-derived suppressor cells, and osteoclast-mediated immunosuppressive circuits [1,139]. These factors not only impair T-cell function but also promote osteolysis and skeletal morbidity. Targeting angiogenic pathways, for instance with VEGF blockade, has been shown to enhance T-cell infiltration and sensitize tumors to immunotherapy [140,141]. Similarly, combining CAR-T with immune checkpoint inhibitors or macrophage reprogramming agents can remodel the bone TME and restore effector function [99,142]. Alternative immune-cell platforms may address some limitations of CAR-T therapy. CAR-NK cells have lower risks of graft-versus-host disease and severe cytokine-mediated toxicity and are more readily suited to allogeneic production [52,143]. Recent EphA2-and CD70-directed CAR-NK studies have shown antitumour activity in osteosarcoma models, although limited persistence and susceptibility to tumour-mediated suppression remain important challenges [51,52]. CAR-M cells offer a different advantage because macrophages naturally infiltrate solid tumors and can combine tumour-cell phagocytosis with modulation of the local immune microenvironment [99]. Preclinical CAR-M studies have demonstrated antigen-specific phagocytosis and recruitment of endogenous T-cell responses, and a first-in-human HER2-CAR-M trial has confirmed manufacturing feasibility, tumour trafficking, and preliminary safety in advanced solid tumors [144]. Thus, CAR-T, CAR-NK, and CAR-M should be viewed as complementary rather than interchangeable platforms: CAR-T currently has the strongest clinical experience and persistence, CAR-NK may be better suited to allogeneic cytotoxic therapy, whereas CAR-M may be particularly useful where tumour infiltration and myeloid-driven immunosuppression are major barriers.

Another limitation is that much of the current preclinical evidence is based on xenograft models in immunodeficient mice. These models are useful for confirming target-dependent CAR-T activity in vivo, but they reproduce only part of the human bone microenvironment. The absence of an intact immune system limits assessment of interactions with myeloid cells, regulatory T cells, and other immune populations that influence CAR-T expansion and persistence. In addition, the stromal and skeletal compartments remain largely murine, including osteoblasts, osteoclasts, marrow stromal cells, vasculature, and extracellular matrix. Subcutaneous xenografts are particularly limited because they do not reproduce the anatomical environment of bone. Future studies should therefore use complementary models where possible. Orthotopic models provide a more relevant skeletal setting and have already been used to evaluate B7-H3 CAR-T cells in osteosarcoma [43]. Immunocompetent models can provide additional information on interactions with an intact immune system, while patient-derived and humanised systems may help preserve tumour heterogeneity or incorporate human cellular components. Three-dimensional culture systems may also be useful for studying antigen heterogeneity and CAR-T penetration before in vivo testing. No single model fully reproduces human bone disease, and findings from conventional xenografts should therefore be confirmed in more physiologically relevant systems before clinical translation.

Safety is a further concern in orthopedics, particularly due to the proximity of tumor antigens to normal mesenchymal or skeletal tissues. HER2 expression in lung epithelium led to fatal toxicity in a sarcoma CAR-T case [36], underscoring the fine balance between efficacy and safety. Although severe CRS is less common than in hematologic malignancies, inflammatory toxicities remain a risk [32]. Safety switches, transient CAR expression, and high-throughput antigen screening tailored to bone and mesenchymal tissues are crucial mitigation tools. Furthermore, early clinical trials of next-generation CAR-T products incorporating logic-gated safety modules or inducible suicide switches have demonstrated significant reductions in off-tumor toxicity [[145], [146], [147]].

These observations suggest that the limited efficacy of CAR-T therapy in orthopedic diseases is unlikely to be explained by a single factor, but rather by the combined effects of structural, vascular, and immunological constraints. Overcoming these barriers therefore requires a shift from incremental optimization toward rational, systems-level CAR-T engineering. As summarized in Fig. 3, emerging strategies focus on logic-gated antigen targeting to mitigate heterogeneity, enhancement of trafficking and infiltration through chemokine receptor engineering or local delivery, reinforcement of CAR-T function within immunosuppressive bone niches, and incorporation of safety and persistence-control modules. Advances in allogeneic platforms [130], multi-target CAR designs [54,135], PDCD1-integrated constructs [137], vascular normalization [140], immune reprogramming [142], and built-in safety modules [[145], [146], [147]] hold promise for addressing these obstacles and establishing CAR-T therapy as a transformative modality in orthopedic oncology and beyond.

**Fig. 3:**
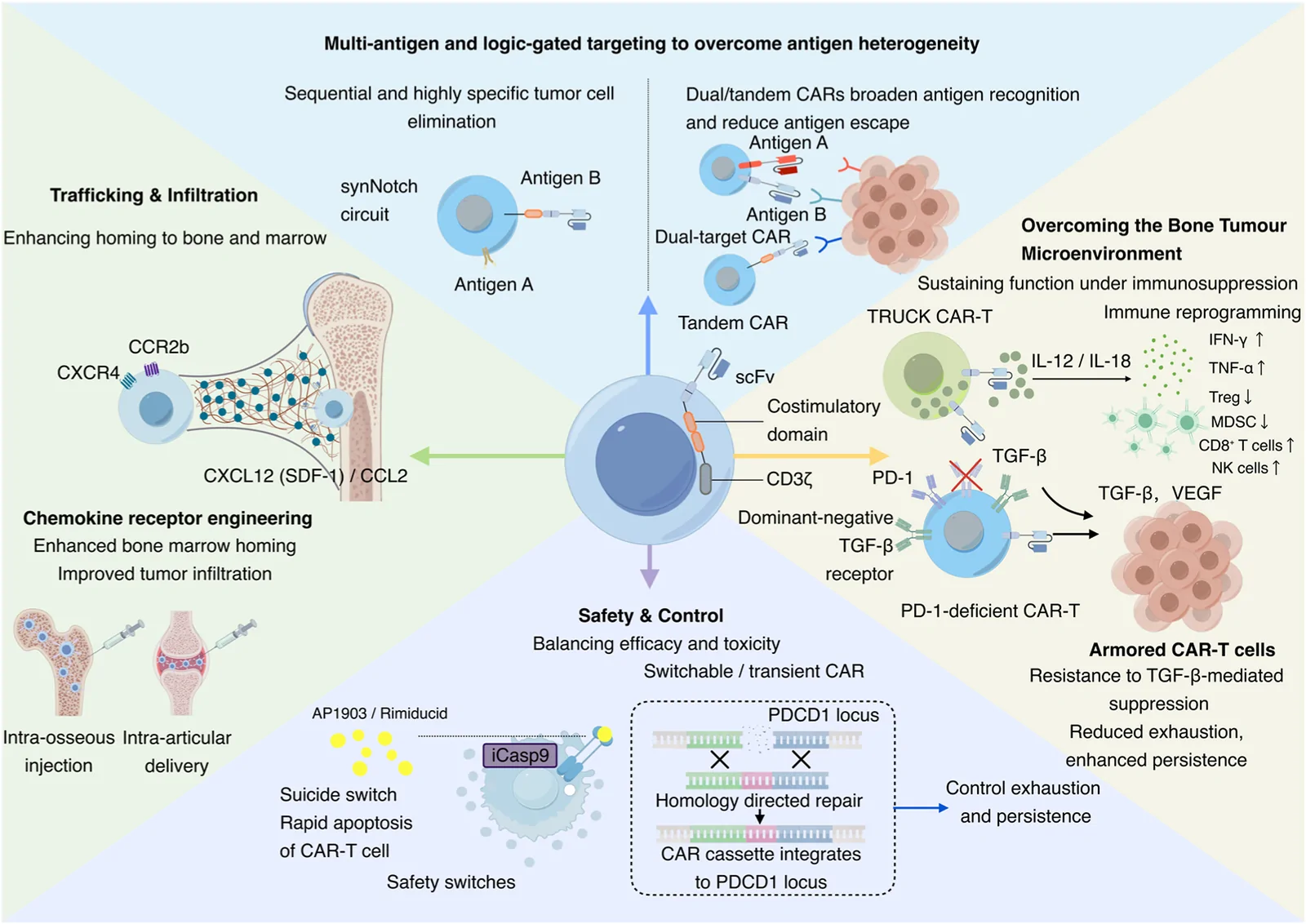
**Engineering strategies to improve CAR-T cell activity in the bone microenvironment.** CAR-T cell function in bone-associated diseases can be enhanced through multiple engineering approaches targeting key barriers. To improve trafficking and infiltration, chemokine receptor engineering (for example, CXCR4 or CCR2b) and local delivery strategies, including intra-osseous and intra-articular administration, are used to promote homing to bone and marrow sites. Antigen heterogeneity is addressed through logic-gated systems, such as synNotch circuits, as well as dual- and tandem-CAR designs, enabling more specific and sequential tumor cell recognition. To counteract immunosuppression, CAR-T cells can be further modified by incorporating cytokine-secreting modules (for example, IL-12 or IL-18), disrupting inhibitory pathways (such as PD-1), or expressing dominant-negative receptors to block TGF-β signalling. Safety and control are achieved through inducible suicide switches (for example, iCasp9) and site-specific genome editing approaches, including targeted integration of CAR constructs into the PDCD1 locus. These strategies aim to enhance CAR-T cell persistence, maintain effector function, and limit treatment-associated toxicity.

## 5 Conclusions and perspective

CAR-T therapy in orthopaedics remains at an early stage of development. In osteosarcoma and other bone malignancies, preclinical and early clinical studies have established feasibility, but objective and durable tumour responses remain uncommon [32,37,61,88]. Antigen heterogeneity, limited trafficking and persistence, and the suppressive bone microenvironment continue to restrict efficacy [1,131,133,136,137]. In non-malignant disease, the evidence is more heterogeneous. CD19-directed CAR-T therapy has begun to show clinical activity in refractory rheumatoid arthritis and other autoimmune rheumatic disorders [109,[119], [120], [121]], whereas senolytic CAR-T approaches for osteoarthritis remain preclinical proof-of-concept strategies [28].

Future development should therefore focus on a limited number of translational priorities. Candidate targets need to provide sufficient disease coverage while maintaining an acceptable normal-tissue safety profile, and promising products should be evaluated in models that better reproduce the immune and skeletal microenvironment [43,54]. For bone malignancies, improving tumour access and maintaining CAR-cell persistence and function within the local microenvironment are likely to be more important than simply increasing receptor signalling strength [91,[136], [137], [138]]. Safety, manufacturing time, and cost will also determine whether these therapies can move beyond highly selected patients [129,130,[145], [146], [147]]. CAR-T is therefore unlikely to replace current orthopaedic treatments in the near term, but it may become a useful option for selected refractory bone malignancies and autoimmune rheumatic disorders if these biological and practical limitations can be addressed.

## Author contributions

All authors contributed significantly to the manuscript. Xugui Li, Yimei Que, Changtian Gong, and Xienilai Xie were responsible for the initial conceptualization and design of the study, led the formal analysis, and prepared the original draft. Wei Xie, Yunzhou Zuo, Wang Chen, and Zedong Xu conducted data curation, investigation, and participated in critical revisions for intellectual content. Xugui Li, Changtian Gong, and Yimei Que provided resources, oversaw project administration, and participated in securing funding for the study. All authors approved the final version of the manuscript for submission. The authors would like to thank all who contributed to this work.

## Declaration of AI and AI-assisted technologies in the writing process

During the preparation of this manuscript, the authors used AI-assisted language tools to improve language clarity and readability. The authors carefully reviewed and edited the content as needed and take full responsibility for the final version of the manuscript.

## Funding statement

This work was supported by the Youth Program of the 10.13039/501100003819Natural Science Foundation of Hubei Province (Grant No. 2025AFB228) and the 10.13039/501100001809National Natural Science Foundation of China (NSFC, Grant No. 82501058).

## Declaration of competing interest

The authors declare no conflict of interest.

## References

[1] Tian H., Cao J., Li B., Nice E.C., Mao H., Zhang Y. (2023). Managing the immune microenvironment of osteosarcoma: the outlook for osteosarcoma treatment. Bone Res.

[2] Gazendam A., Popovic S., Parasu N., Ghert M. (2023). Chondrosarcoma: a clinical review. J Clin Med.

[3] Yang W., Pan Q., Huang F., Hu H., Shao Z. (2022). Research progress of bone metastases: from disease recognition to clinical practice. Front Oncol.

[4] Yu S., Yao X. (2024). Advances on immunotherapy for osteosarcoma. Mol Cancer.

[5] Xu Y., Wu Q. (2021). Prevalence trend and disparities in rheumatoid arthritis among us adults, 2005-2018. J Clin Med.

[6] Madden D.L. (2018). From a patient advocate's perspective: does cancer immunotherapy represent a paradigm shift. Curr Oncol Rep.

[7] Yoo H.J., Harapan B.N. (2021). Chimeric antigen receptor (CAR) immunotherapy: basic principles, current advances, and future prospects in neuro-oncology. Immunol Res.

[8] Sterner R.C., Sterner R.M. (2021). CAR-T cell therapy: current limitations and potential strategies. Blood Cancer J.

[9] June C.H., Sadelain M. (2018). Chimeric antigen receptor therapy. N Engl J Med.

[10] Philipson B.I., O'Connor R.S., May M.J., June C.H., Albelda S.M., Milone M.C. (2020). 4-1BB costimulation promotes CAR-T cell survival through noncanonical NF-κB signaling. Sci Signal.

[11] Roselli E., Boucher J.C., Li G., Kotani H., Spitler K., Reid K. (2021). 4-1BB and optimized CD28 co-stimulation enhances function of human mono-specific and bi-specific third-generation CAR-T cells. J Immunother Cancer.

[12] Cappell K.M., Kochenderfer J.N. (2023). Long-term outcomes following CAR-T cell therapy: what we know so far. Nat Rev Clin Oncol.

[13] Laetsch T.W., Maude S.L., Rives S., Hiramatsu H., Bittencourt H., Bader P. (2023). Three-year update of tisagenlecleucel in pediatric and young adult patients with relapsed/refractory acute lymphoblastic leukemia in the eliana trial. J Clin Oncol.

[14] Shah B.D., Ghobadi A., Oluwole O.O., Logan A.C., Boissel N., Cassaday R.D. (2021). Kte-x19 for relapsed or refractory adult b-cell acute lymphoblastic leukaemia: phase 2 results of the single-arm, open-label, multicentre zuma-3 study. Lancet.

[15] Munshi N.C., Anderson L.J., Shah N., Madduri D., Berdeja J., Lonial S. (2021). Idecabtagene vicleucel in relapsed and refractory multiple myeloma. N Engl J Med.

[16] Berdeja J.G., Madduri D., Usmani S.Z., Jakubowiak A., Agha M., Cohen A.D. (2021). Ciltacabtagene autoleucel, a b-cell maturation antigen-directed chimeric antigen receptor t-cell therapy in patients with relapsed or refractory multiple myeloma (cartitude-1): a phase 1b/2 open-label study. Lancet.

[17] Jagannath S., Martin T.G., Lin Y., Cohen A.D., Raje N., Htut M. (2025). Long-term (>/=5-year) remission and survival after treatment with ciltacabtagene autoleucel in cartitude-1 patients with relapsed/refractory multiple myeloma. J Clin Oncol.

[18] Huynh T.H.N., Clampitt B.A., Chose C.M., Nester M.D., Joyce D.M., Binitie O.T. (2026). Influence of chimeric antigen receptor t-cell therapy on fracture risk of patients with multiple myeloma. J Am Acad Orthop Surg.

[19] Okamoto K. (2024). Crosstalk between bone and the immune system. J Bone Miner Metabol.

[20] Deng Z., Zhang Q., Zhao Z., Li Y., Chen X., Lin Z. (2021). Crosstalk between immune cells and bone cells or chondrocytes. Int Immunopharmacol.

[21] Horas K., Menale C., Maurizi A. (2022). Editorial: the bone/bone marrow microenvironment: a hub for immune regulation of the tumor cells fate. Front Immunol.

[22] Peng Z., Huang W., Xiao Z., Wang J., Zhu Y., Zhang F. (2024). Immunotherapy in the fight against bone metastases: a review of recent developments and challenges. Curr Treat Options Oncol.

[23] Knights A.J., Redding S.J., Maerz T. (2023). Inflammation in osteoarthritis: the latest progress and ongoing challenges. Curr Opin Rheumatol.

[24] Jang S., Kwon E., Lee J.J. (2022). Rheumatoid arthritis: pathogenic roles of diverse immune cells. Int J Mol Sci.

[25] Kankeu Fonkoua L.A., Sirpilla O., Sakemura R., Siegler E.L., Kenderian S.S. (2022). CAR-T cell therapy and the tumor microenvironment: current challenges and opportunities. Mol Ther Oncolytics.

[26] Liu G., Rui W., Zhao X., Lin X. (2021). Enhancing CAR-T cell efficacy in solid tumors by targeting the tumor microenvironment. Cell Mol Immunol.

[27] Bughda R., Dimou P., D'Souza R.R., Klampatsa A. (2021). Fibroblast activation protein (fap)-targeted CAR-T cells: launching an attack on tumor stroma. ImmunoTargets Ther.

[28] Amor C., Feucht J., Leibold J., Ho Y., Zhu C., Alonso-Curbelo D. (2020). Senolytic CAR-T cells reverse senescence-associated pathologies. Nature.

[29] Joyce J.A., Fearon D.T. (2015). T cell exclusion, immune privilege, and the tumor microenvironment. Science (New York, NY).

[30] Wherry E.J., Kurachi M. (2015). Molecular and cellular insights into T cell exhaustion. Nat Rev Immunol.

[31] Ahmed N., Brawley V.S., Hegde M., Robertson C., Ghazi A., Gerken C. (2015). Human epidermal growth factor receptor 2 (HER2) -specific chimeric antigen receptor-modified T cells for the immunotherapy of HER2-positive sarcoma. J Clin Oncol.

[32] Hegde M., Navai S., DeRenzo C., Joseph S.K., Sanber K., Wu M. (2024). Autologous HER2-specific CAR-T cells after lymphodepletion for advanced sarcoma: a phase 1 trial. Nat Cancer.

[33] Yarden Y., Sliwkowski M.X. (2001). Untangling the erbb signalling network. Nat Rev Mol Cell Biol.

[34] Morris C.D., Gorlick R., Huvos G., Heller G., Meyers P.A., Healey J.H. (2001). Human epidermal growth factor receptor 2 as a prognostic indicator in osteogenic sarcoma. Clin Orthop Relat Res.

[35] Reed D.R.A.J. (2025). Pepn1924, a phase ii study of trastuzumab deruxtecan in patients with recurrent human epidermal growth factor receptor 2+ osteosarcoma: a children's oncology group pediatric early-phase clinical trial network study. JCO Oncol Adv.

[36] Morgan R.A., Yang J.C., Kitano M., Dudley M.E., Laurencot C.M., Rosenberg S.A. (2010). Case report of a serious adverse event following the administration of T cells transduced with a chimeric antigen receptor recognizing erbb2. Mol Ther : the journal of the American Society of Gene Therapy.

[37] Kaczanowska S., Murty T., Alimadadi A., Contreras C.F., Duault C., Subrahmanyam P.B. (2024). Immune determinants of CAR-T cell expansion in solid tumor patients receiving GD2 CAR-T cell therapy. Cancer Cell.

[38] Yankelevich M., Thakur A., Modak S., Chu R., Taub J., Martin A. (2024). Targeting refractory/recurrent neuroblastoma and osteosarcoma with anti-CD3×anti-GD2 bispecific antibody armed T cells. J Immunother Cancer.

[39] Fernández L., Metais J., Escudero A., Vela M., Valentín J., Vallcorba I. (2017). Memory T cells expressing an NKG2D-CAR efficiently target osteosarcoma cells. Clin Cancer Res.

[40] Hidalgo L., Garcia-Rodriguez P., Cubillo I., Zubizarreta M., Perez-Martinez A., García-Castro J. (2026). Unaltered NKG2D-CAR T cell function under hypoxia in osteosarcoma in vitro. Cancer Immunol Immunother.

[41] Lynch M.M., Al-Marayaty R., Obeidin F., Alexiev B.A., Chen E.Y., Viveiros P. (2024). B7-H3 is widely expressed in soft tissue sarcomas. BMC Cancer.

[42] Majzner R.G., Theruvath J.L., Nellan A., Heitzeneder S., Cui Y., Mount C.W. (2019). CAR-T cells targeting B7-H3, a pan-cancer antigen, demonstrate potent preclinical activity against pediatric solid tumors and brain tumors. Clin Cancer Res.

[43] Talbot L.J., Chabot A., Funk A., Nguyen P., Wagner J., Ross A. (2021). A novel orthotopic implantation technique for osteosarcoma produces spontaneous metastases and illustrates dose-dependent efficacy of B7-H3 CAR-T cells. Front Immunol.

[44] Mensali N., Köksal H., Joaquina S., Wernhoff P., Casey N.P., Romecin P. (2023). Alpl-1 is a target for chimeric antigen receptor therapy in osteosarcoma. Nat Commun.

[45] Wang Y., Yu W., Zhu J., Wang J., Xia K., Liang C. (2019). Anti-CD166/4-1BB chimeric antigen receptor T cell therapy for the treatment of osteosarcoma. J Exp Clin Cancer Res.

[46] Wang L.S., Lo A., Scholler J., Sun J., Majumdar R.S., Kapoor V. (2014). Targeting fibroblast activation protein in tumor stroma with chimeric antigen receptor T cells can inhibit tumor growth and augment host immunity without severe toxicity. Cancer Immunol Res.

[47] Riccardo F., Tarone L., Iussich S., Giacobino D., Arigoni M., Sammartano F. (2019). Identification of CSPG4 as a promising target for translational combinatorial approaches in osteosarcoma. Ther Adv Med Oncol.

[48] Feinberg D., Uppin V., Eid S., Fritz C., Hitomi M., Radhakrishnan A. (2026). Oncostatin-m ligand-based CAR-T therapy displays robust anti-tumor activity against osteosarcoma. BMC Med.

[49] Choe M., Kirkey D., Lira I., Hawkins G., Blankenfeld M., Menashe S. (2025). Preclinical evaluation of folate receptor-α chimeric antigen receptor T cells exhibits highly efficient antitumor activity against osteosarcoma. Cancer Res Commun.

[50] O'Reilly C., Tuladhar S., Nguyen P., Sheppard H., Koo S.C., Chockley P. (2026). LRRC15-CAR-T cells for the treatment of osteosarcoma. Clin Cancer Res.

[51] Lam P.Y., Omer N., Wong J.K.M., Tu C., Alim L., Rossi G.R. (2025). Enhancement of anti-sarcoma immunity by nk cells engineered with mRNA for expression of a EphA2-targeted CAR. Clin Transl Med.

[52] Robbins G.M., Chang J., Krueger J.B., Vue Y.Y., Gilkey A.K., Folsom T.D. (2025). Non-viral TcBuster transposon engineering of CD70-CAR natural killer cells for the treatment of osteosarcoma. Mol Ther Oncol.

[53] Huang G., Yu L., Cooper L.J., Hollomon M., Huls H., Kleinerman E.S. (2012). Genetically modified T cells targeting interleukin-11 receptor α-chain kill human osteosarcoma cells and induce the regression of established osteosarcoma lung metastases. Cancer Res.

[54] Roybal K.T., Rupp L.J., Morsut L., Walker W.J., McNally K.A., Park J.S. (2016). Precision tumor recognition by T cells with combinatorial antigen-sensing circuits. Cell.

[55] Cheng S., Wang H., Kang X., Zhang H. (2024). Immunotherapy innovations in the fight against osteosarcoma: emerging strategies and promising progress. Pharmaceutics.

[56] Gelderblom H., Hogendoorn P.C.W., Dijkstra S.D., van Rijswijk C.S., Krol A.D., Taminiau A.H.M. (2008). The clinical approach towards chondrosarcoma. Oncologist.

[57] Strauss S.J., Frezza A.M., Abecassis N., Bajpai J., Bauer S., Biagini R. (2021). Bone sarcomas: esmo-euracan-genturis-ern paedcan clinical practice guideline for diagnosis, treatment and follow-up. Ann Oncol.

[58] Tap W.D., Villalobos V.M., Cote G.M., Burris H., Janku F., Mir O. (2020). Phase i study of the mutant idh1 inhibitor ivosidenib: safety and clinical activity in patients with advanced chondrosarcoma. J Clin Oncol.

[59] Chow W., Frankel P., Ruel C., Araujo D.M., Milhem M., Okuno S. (2020). Results of a prospective phase 2 study of pazopanib in patients with surgically unresectable or metastatic chondrosarcoma. Cancer.

[60] Lu V.M., O'Connor K.P., Mahajan A., Carlson M.L., Van Gompel J.J. (2020). Carbon ion radiotherapy for skull base chordomas and chondrosarcomas: a systematic review and meta-analysis of local control, survival, and toxicity outcomes. J Neuro Oncol.

[61] Nota S.P.F.T., Osei-Hwedieh D.O., Drum D.L., Wang X., Sabbatino F., Ferrone S. (2022). Chondroitin sulfate proteoglycan 4 expression in chondrosarcoma: a potential target for antibody-based immunotherapy. Front Oncol.

[62] Jamil N.S.M., Azfer A., Worrell H., Salter D.M. (2016). Functional roles of cspg4/ng2 in chondrosarcoma. Int J Exp Pathol.

[63] Wang X., Osada T., Wang Y., Yu L., Sakakura K., Katayama A. (2010). Cspg4 protein as a new target for the antibody-based immunotherapy of triple-negative breast cancer. J Natl Cancer Inst.

[64] Hsu N.C., Nien P., Yokoyama K.K., Chu P., Hou M. (2013). High chondroitin sulfate proteoglycan 4 expression correlates with poor outcome in patients with breast cancer. Biochem Biophys Res Commun.

[65] Hesla A.C., Papakonstantinou A., Tsagkozis P. (2021). Current status of management and outcome for patients with ewing sarcoma. Cancers.

[66] Rasper M., Jabar S., Ranft A., Jürgens H., Amler S., Dirksen U. (2014). The value of high-dose chemotherapy in patients with first relapsed ewing sarcoma. Pediatr Blood Cancer.

[67] Womer R.B., West D.C., Krailo M.D., Dickman P.S., Pawel B.R., Grier H.E. (2012). Randomized controlled trial of interval-compressed chemotherapy for the treatment of localized ewing sarcoma: a report from the children's oncology group. J Clin Oncol.

[68] Kulczycka M., Derlatka K., Tasior J., Lejman M., Zawitkowska J. (2023). CAR-T cell therapy in children with solid tumors. J Clin Med.

[69] Ravanpay A.C., Gust J., Johnson A.J., Rolczynski L.S., Cecchini M., Chang C.A. (2019). EGFR806 CAR-T cells selectively target a tumor-restricted egfr epitope in glioblastoma. Oncotarget.

[70] Lehner M., Götz G., Proff J., Schaft N., Dörrie J., Full F. (2012). Redirecting T cells to ewing's sarcoma family of tumors by a chimeric NKG2D receptor expressed by lentiviral transduction or mRNA transfection. PLoS One.

[71] Garcia-Monclús S., López-Alemany R., Almacellas-Rabaiget O., Herrero-Martín D., Huertas-Martinez J., Lagares-Tena L. (2018). EphA2 receptor is a key player in the metastatic onset of ewing sarcoma. Int J Cancer.

[72] Hsu K., Middlemiss S., Saletta F., Gottschalk S., McCowage G.B., Kramer B. (2021). Chimeric antigen receptor-modified T cells targeting EphA2 for the immunotherapy of paediatric bone tumours. Cancer Gene Ther.

[73] Pappo A.S., Patel S.R., Crowley J., Reinke D.K., Kuenkele K., Chawla S.P. (2011). R1507, a monoclonal antibody to the insulin-like growth factor 1 receptor, in patients with recurrent or refractory ewing sarcoma family of tumors: results of a phase ii sarcoma alliance for research through collaboration study. J Clin Oncol.

[74] Tap W.D., Demetri G., Barnette P., Desai J., Kavan P., Tozer R. (2012). Phase ii study of ganitumab, a fully human anti-type-1 insulin-like growth factor receptor antibody, in patients with metastatic ewing family tumors or desmoplastic small round cell tumors. J Clin Oncol.

[75] Naing A., LoRusso P., Fu S., Hong D.S., Anderson P., Benjamin R.S. (2012). Insulin growth factor-receptor (IGF-1R) antibody cixutumumab combined with the mTOR inhibitor temsirolimus in patients with refractory ewing's sarcoma family tumors. Clin Cancer Res.

[76] Schwartz G.K., Tap W.D., Qin L., Livingston M.B., Undevia S.D., Chmielowski B. (2013). Cixutumumab and temsirolimus for patients with bone and soft-tissue sarcoma: a multicentre, open-label, phase 2 trial. Lancet Oncol.

[77] Strauss S.J., Berlanga P., McCabe M.G. (2024). Emerging therapies in ewing sarcoma. Curr Opin Oncol.

[78] Coleman R.E., Croucher P.I., Padhani A.R., Clézardin P., Chow E., Fallon M. (2020). Bone metastases. Nat Rev Dis Primers.

[79] Saad F., Gleason D.M., Murray R., Tchekmedyian S., Venner P., Lacombe L. (2002). A randomized, placebo-controlled trial of zoledronic acid in patients with hormone-refractory metastatic prostate carcinoma. J Natl Cancer Inst.

[80] Stopeck A.T., Lipton A., Body J., Steger G.G., Tonkin K., de Boer R.H. (2010). Denosumab compared with zoledronic acid for the treatment of bone metastases in patients with advanced breast cancer: a randomized, double-blind study. J Clin Oncol.

[81] Fizazi K., Carducci M., Smith M., Damião R., Brown J., Karsh L. (2011). Denosumab versus zoledronic acid for treatment of bone metastases in men with castration-resistant prostate cancer: a randomised, double-blind study. Lancet (London, England).

[82] Parker C., Nilsson S., Heinrich D., Helle S.I., O'Sullivan J.M., Foss S.D. (2013). Alpha emitter radium-223 and survival in metastatic prostate cancer. N Engl J Med.

[83] Yao Y., Zheng X., Luo X., Wu A. (2021). Incidence, prognosis and nomograms of breast cancer with bone metastases at initial diagnosis: a large population-based study. Am J Transl Res.

[84] Sugiura H., Yamada K., Sugiura T., Hida T., Mitsudomi T. (2008). Predictors of survival in patients with bone metastasis of lung cancer. Clin Orthop Relat Res.

[85] Zhu Y., She J., Sun R., Yan X., Huang X., Wang P. (2024). Impact of bone metastasis on prognosis in non-small cell lung cancer patients treated with immune checkpoint inhibitors: a systematic review and meta-analysis. Front Immunol.

[86] Gu Z., Thomas G., Yamashiro J., Shintaku I.P., Dorey F., Raitano A. (2000). Prostate stem cell antigen (PSCA) expression increases with high gleason score, advanced stage and bone metastasis in prostate cancer. Oncogene.

[87] Lam J.S., Yamashiro J., Shintaku I.P., Vessella R.L., Jenkins R.B., Horvath S. (2005). Prostate stem cell antigen is overexpressed in prostate cancer metastases. Clin Cancer Res.

[88] Dorff T.B., Blanchard M.S., Adkins L.N., Luebbert L., Leggett N., Shishido S.N. (2024). PSCA CAR-T cell therapy in metastatic castration-resistant prostate cancer: a phase 1 trial. Nat Med.

[89] Seitz C.M., Schroeder S., Knopf P., Krahl A., Hau J., Schleicher S. (2019). GD2-targeted chimeric antigen receptor T cells prevent metastasis formation by elimination of breast cancer stem-like cells. Oncoimmunology.

[90] Reppel L.C., Tsahouridis O., Akulian J., Davis I.J., Lee H., Fucà G. (2022). Targeting disialoganglioside GD2 with chimeric antigen receptor-redirected T cells in lung cancer. J Immunother Cancer.

[91] Khan A.B., Carpenter B., Santos E., Sousa P., Pospori C., Khorshed R. (2018). Redirection to the bone marrow improves T cell persistence and antitumor functions. J Clin Investig.

[92] Curio S., Jonsson G., Marinovi S. (2021). A summary of current NKG2D-based CAR clinical trials. Immunother Adv.

[93] Tang C., Lai Y., Li L., Situ M., Li S., Cheng B. (2024). Serpinh1 modulates apoptosis by inhibiting p62 ubiquitination degradation to promote bone metastasis of prostate cancer. iScience.

[94] He B., Ganss R. (2021). Modulation of the vascular-immune environment in metastatic cancer. Cancers.

[95] He N., Jiang J. (2022). Contribution of immune cells to bone metastasis pathogenesis. Front Endocrinol.

[96] Sousa S., Määttä J. (2016). The role of tumour-associated macrophages in bone metastasis. J Bone Oncol.

[97] Joseph G.J., Johnson D.B., Johnson R.W. (2023). Immune checkpoint inhibitors in bone metastasis: clinical challenges, toxicities, and mechanisms. J Bone Oncol.

[98] Jain R.K. (2014). Antiangiogenesis strategies revisited: from starving tumors to alleviating hypoxia. Cancer Cell.

[99] Klichinsky M., Ruella M., Shestova O., Lu X.M., Best A., Zeeman M. (2020). Human chimeric antigen receptor macrophages for cancer immunotherapy. Nat Biotechnol.

[100] Taylor P.C., Matucci Cerinic M., Alten R., Avouac J.M., Westhovens R. (2022). Managing inadequate response to initial anti-tnf therapy in rheumatoid arthritis: optimising treatment outcomes. Ther Adv Musculoskel.

[101] Nozaki Y., Kishimoto K., Tomita D., Itami T., Ashida C., Shiga T. (2025). Stepwise dose reduction and discontinuation of bdmard in rheumatoid arthritis: a prospective cohort study of flare-free population, flares, and predictive markers. Arthritis Res Ther.

[102] Qiu Q., Huang J., Shu X., Fan H., Zhou Y., Xiao C. (2017). Polymorphisms and pharmacogenomics for the clinical efficacy of methotrexate in patients with rheumatoid arthritis: a systematic review and meta-analysis. Sci Rep-Uk.

[103] Kamburova E.G., Koenen H.J.P.M., Borgman K.J.E., Ten Berge I.J., Joosten I., Hilbrands L.B. (2013). A single dose of rituximab does not deplete b cells in secondary lymphoid organs but alters phenotype and function. Am J Transplant.

[104] Li Y., Lyu Z., Chen Y., Fang Y., Yang L. (2024). Frontiers in CAR-T cell therapy for autoimmune diseases. Trends Pharmacol Sci.

[105] Mohseni Y.R., Tung S.L., Dudreuilh C., Lechler R.I., Fruhwirth G.O., Lombardi G. (2020). The future of regulatory T cell therapy: promises and challenges of implementing CAR technology. Front Immunol.

[106] Zhang B., Wang Y., Yuan Y., Sun J., Liu L., Huang D. (2021). In vitro elimination of autoreactive b cells from rheumatoid arthritis patients by universal chimeric antigen receptor T cells. Ann Rheum Dis.

[107] Dall'Era M., Pauli M.L., Remedios K., Taravati K., Sandova P.M., Putnam A.L. (2019). Adoptive treg cell therapy in a patient with systemic lupus erythematosus. Arthritis Rheumatol.

[108] Whittington K.B., Prislovsky A., Beaty J., Albritton L., Radic M., Rosloniec E.F. (2022). CD8(+) T cells expressing an HLA-DR1 chimeric antigen receptor target autoimmune CD4(+) T cells in an antigen-specific manner and inhibit the development of autoimmune arthritis. J Immunol.

[109] Lidar M., Rimar D., David P., Jacoby E., Shapira-Frommer R., Itzhaki O. (2025). CD-19 CAR-T cells for polyrefractory rheumatoid arthritis. Ann Rheum Dis.

[110] Brown S., Kumar S., Sharma B. (2019). Intra-articular targeting of nanomaterials for the treatment of osteoarthritis. Acta Biomater.

[111] Wen J., Li H., Dai H., Hua S., Long X., Li H. (2023). Intra-articular nanoparticles based therapies for osteoarthritis and rheumatoid arthritis management. Mater Today Bio.

[112] Lu L., Lan Q., Li Z., Zhou X., Gu J., Li Q. (2014). Critical role of all-trans retinoic acid in stabilizing human natural regulatory T cells under inflammatory conditions. P Natl Acad Sci Usa.

[113] Barbi J., Pardoll D., Pan F. (2014). Treg functional stability and its responsiveness to the microenvironment. Immunol Rev.

[114] Lu L., Xie M., Yang B., Zhao W., Cao J. (2024). Enhancing the safety of CAR-T cell therapy: synthetic genetic switch for spatiotemporal control. Sci Adv.

[115] Park H.B., Kim K.H., Kim J.H., Kim S.I., Oh Y.M., Kang M. (2024). Improved safety of chimeric antigen receptor T cells indirectly targeting antigens via switchable adapters. Nat Commun.

[116] Shah P.D., Huang A.C., Xu X., Orlowski R., Amaravadi R.K., Schuchter L.M. (2023). Phase I trial of autologous RNA-electroporated CMET-directed CAR-T cells administered intravenously in patients with melanoma and breast carcinoma. Cancer Res Commun.

[117] Chikanza I.C., Sakkas L.I. (2026). Chimeric antigen receptor macrophage (CAR-M) immunotherapy in autoimmune inflammatory rheumatic diseases. Clin Rheumatol.

[118] Sundbaum J.K., Baecklund E., Eriksson N., Hallberg P.R., Kohnke H., Wadelius M. (2020). Mthfr, tyms and slco1b1 polymorphisms and adverse liver effects of methotrexate in rheumatoid arthritis. Pharmacogenomics.

[119] Müller F., Taubmann J., Bucci L., Wilhelm A., Bergmann C., Völkl S. (2024). CD19 CAR-T cell therapy in autoimmune disease - a case series with follow-up. N Engl J Med.

[120] Auth J., Müller F., Völkl S., Bayerl N., Distler J.R.H.W., Tur C. (2025). CD19-targeting CAR-T cell therapy in patients with diffuse systemic sclerosis: a case series. The Lancet. Rheumatology.

[121] Wang X., Wu X., Tan B., Zhu L., Zhang Y., Lin L. (2024). Allogeneic CD19-targeted CAR-T therapy in patients with severe myositis and systemic sclerosis. Cell.

[122] Hunter D.J., Bierma-Zeinstra S. (2019). Osteoarthritis. Lancet (London, England).

[123] Moulin D., Sellam J., Berenbaum F., Guicheux J.M., Boutet M. (2025). The role of the immune system in osteoarthritis: mechanisms, challenges and future directions. Nat Rev Rheumatol.

[124] Loeser R.F., Collins J.A., Diekman B.O. (2016). Ageing and the pathogenesis of osteoarthritis. Nat Rev Rheumatol.

[125] Liu Y., Zhang Z., Li T., Xu H., Zhang H. (2022). Senescence in osteoarthritis: from mechanism to potential treatment. Arthritis Res Ther.

[126] Jeon O.H., Kim C., Laberge R., Demaria M., Rathod S., Vasserot A.P. (2017). Local clearance of senescent cells attenuates the development of post-traumatic osteoarthritis and creates a pro-regenerative environment. Nat Med.

[127] Demaria M., Ohtani N., Youssef S.A., Rodier F., Toussaint W., Mitchell J.R. (2014). An essential role for senescent cells in optimal wound healing through secretion of PDGF-AA. Dev Cell.

[128] Ottaviani G., Jaffe N. (2009). The epidemiology of osteosarcoma. Cancer Treat Res.

[129] Fiorenza S., Ritchie D.S., Ramsey S.D., Turtle C.J., Roth J.A. (2020). Value and affordability of CAR t-cell therapy in the United States. Bone Marrow Transplant.

[130] Chen S., van den Brink M.R.M. (2024). Allogeneic “off-the-shelf” CAR-T cells: challenges and advances. Best Pract Res Clin Haematol.

[131] Wiebel M., Kailayangiri S., Altvater B., Meltzer J., Grobe K., Kupich S. (2021). Surface expression of the immunotherapeutic target g(d2) in osteosarcoma depends on cell confluency. Cancer Rep (Hoboken).

[132] Lin H., Yang X., Ye S., Huang L., Mu W. (2024). Antigen escape in CAR-T cell therapy: mechanisms and overcoming strategies. Biomed Pharmacother.

[133] Zhu J., Simayi N., Wan R., Huang W. (2022). CAR-T targets and microenvironmental barriers of osteosarcoma. Cytotherapy.

[134] Hyrenius-Wittsten A., Su Y., Park M., Garcia J.M., Alavi J., Perry N. (2021). Synnotch CAR circuits enhance solid tumor recognition and promote persistent antitumor activity in mouse models. Sci Transl Med.

[135] Hegde M., Mukherjee M., Grada Z., Pignata A., Landi D., Navai S.A. (2021). Tandem CAR-T cells targeting HER2 and il13rα2 mitigate tumor antigen escape. J Clin Investig.

[136] Craddock J.A., Lu A., Bear A., Pule M., Brenner M.K., Rooney C.M. (2010). Enhanced tumor trafficking of GD2 chimeric antigen receptor T cells by expression of the chemokine receptor CCR2b. J Immunother.

[137] Hu Y., Zu C., Zhang M., Wei G., Li W., Fu S. (2023). Safety and efficacy of CRISPR-based non-viral PD-1 locus specifically integrated anti-CD19 CAR-T cells in patients with relapsed or refractory non-hodgkin's lymphoma: a first-in-human phase i study. EClinicalMedicine.

[138] Sagnella S.M., White A.L., Yeo D., Saxena P., van Zandwijk N., Rasko J.E.J. (2022). Locoregional delivery of CAR-T cells in the clinic. Pharmacol Res.

[139] Russo S., Scotto Di Carlo F., Gianfrancesco F. (2022). The osteoclast traces the route to bone tumors and metastases. Front Cell Dev Biol.

[140] Huang Y., Yuan J., Righi E., Kamoun W.S., Ancukiewicz M., Nezivar J. (2012). Vascular normalizing doses of antiangiogenic treatment reprogram the immunosuppressive tumor microenvironment and enhance immunotherapy. P Natl Acad Sci Usa.

[141] Park J.A., Espinosa-Cotton M., Guo H., Monette S., Cheung N.V. (2023). Targeting tumor vasculature to improve antitumor activity of T cells armed ex vivo with T cell engaging bispecific antibody. J Immunother Cancer.

[142] Grosser R., Cherkassky L., Chintala N., Adusumilli P.S. (2019). Combination immunotherapy with CAR-T cells and checkpoint blockade for the treatment of solid tumors. Cancer Cell.

[143] Zuo S., Cheng N., Hou Z., Yang Y., Tian Q., Xu Y. (2026). Reprogramming adoptive cell therapy for osteosarcoma: engineering, vaccination, and tumor microenvironment remodeling. Front Immunol.

[144] Reiss K.A., Angelos M.G., Dees E.C., Yuan Y., Ueno N.T., Pohlmann P.R. (2025). CAR-macrophage therapy for HER2-overexpressing advanced solid tumors: a phase 1 trial. Nat Med.

[145] Lin H., Cheng J., Zhu L., Zeng Y., Dai Z., Zhang Y. (2024). Anti-CD5 CAR-T cells with a tegfr safety switch exhibit potent toxicity control. Blood Cancer J.

[146] Wermke M., Kraus S., Ehninger A., Bargou R.C., Goebeler M., Middeke J.M. (2021). Proof of concept for a rapidly switchable universal CAR-T platform with uniCAR-T-CD123 in relapsed/refractory aml. Blood.

[147] Di Stasi A., Tey S., Dotti G., Fujita Y., Kennedy-Nasser A., Martinez C. (2011). Inducible apoptosis as a safety switch for adoptive cell therapy. N Engl J Med.

