# Supplementary material for: Application of CAR-T cell therapy in orthopedics: From bone malignancies to non-malignant musculoskeletal disorders
Source: J Orthop Translat. 2026 Oct 10;61:101235. doi: 10.1016/j.jot.2026.101235 (PMC13677999; doi:10.1016/j.jot.2026.101235)
Supplement: Supplementary file 1 [file mmc1.docx]

**Supplementary Table S1. Comparative framework for selected CAR-T targets in bone sarcomas**

| **Target** | **Main indication** | **Tumour expression / heterogeneity** | **Normal-tissue / safety consideration** | **Evidence level** | **Main translational consideration** |
| --- | --- | --- | --- | --- | --- |
| **HER2** | Osteosarcoma | Variable expression among tumours | Normal-tissue expression requires a carefully defined safety window | Clinical | Clinical experience is available, but heterogeneous expression and potential on-target/off-tumour toxicity remain concerns |
| **GD2** | Osteosarcoma; Ewing sarcoma | Expression may vary between tumours | Manageable toxicity reported in early clinical studies | Clinical | Initial disease control has been observed, but responses have generally lacked durability |
| **B7-H3** | Osteosarcoma and other sarcomas | Expression reported across sarcomas; activity demonstrated in OS models | Clinical safety in bone sarcoma remains to be established | Preclinical | Strong antitumour activity in OS models supports further clinical evaluation |
| **ALPL-1** | Osteosarcoma | High and relatively selective expression reported in primary and metastatic OS | Favourable safety profile in preclinical models | Preclinical | Promising tumour selectivity, including metastatic OS, but clinical validation is lacking |
| **CSPG4** | Osteosarcoma; chondrosarcoma | Expression varies between tumours and chondrosarcoma subtypes | Restricted but not absent normal-tissue expression | Preclinical | Useful for selected CSPG4-positive tumours, but patient selection and further safety profiling are required |
| **IL-11Rα** | Osteosarcoma | Expression sufficient for targeting in preclinical OS models | Human safety window remains uncertain | Preclinical | Regression of pulmonary metastatic OS has been demonstrated preclinically, but evidence remains early |
